# Two-Photodetector In-Phase and Quadrature Demodulation Interferometer Scheme Using Temporally Shifted Differential Signals

**DOI:** 10.3390/s26216967

**Published:** 2026-11-04

**Authors:** June Gyu Park, Kyuman Cho

**Affiliations:** 1 Department of Astronomy, Yonsei University, 50 Yonsei-ro, Seodaemun-gu, Seoul 03722, Republic of Korea; j.park@yonsei.ac.kr; 2 Department of Physics, Sogang University, Seoul 04107, Republic of Korea

**Keywords:** homodyne interferometer, in phase quadrature (I-Q) interferometer, phase demodulation, temporal difference, two port interferometer

## Abstract

Four photodetectors, two in the balanced homodyne detector for in-phase (I) channel measurements and two in that for quadrature (Q) channel measurements, are needed for a conventional homodyne I/Q interferometer. Here, we present a new homodyne I/Q interferometer scheme for which two photodetectors, one for I-channel and the other for Q-channel measurements, are used for I/Q demodulation of the interference signal. The DC bias terms on the output signal from each photodetector are rejected by taking the difference between the signal and the temporally shifted signal. Then the induced phase on the interference signal can be measured by using the algorithm for I/Q phase demodulation. It will be shown that the sensitivity is limited by the corresponding photon noise. We will also show that in the vicinity of the turning points of a periodic modulation, where the phase difference may become smaller than the corresponding photon noise, or in the region where there is no measurable phase change the phase can be calculated through temporal averaging, which not only removes high-frequency noises but also increases the time delay for obtaining the difference signal.

## 1. Introduction

Two-beam interferometer (TBI) schemes, either homodyne or heterodyne, have been widely implemented for high-sensitivity sensor applications across various scientific and engineering disciplines [1,2,3,4,5,6,7,8]. They have been used for measuring extremely small phase and/or amplitude changes between two arms, which are limited by the corresponding photon noise [9,10,11,12,13]. For a conventional homodyne TBI, depending on the applications, the phase difference between the two arms of the interferometer is locked at the specific value to keep the TBI at either a dark port or a quadrature demodulation condition by using feedback control of the length of one arm [11,12,13,14,15,16,17]. Then, the output signal is monitored to measure a small change in the output signal caused by the corresponding optical change of either the phase or the amplitude: it can be used for measuring an extremely small change in optical path length or a very-weak-intensity beam such as a stream of single photons. Both arms can be used as an antenna, for example, of a gravitational wave detector or for a sensor application, where one arm is used as a probe or a signal beam while the other arm is used as a reference beam or a local oscillator. Since the change in the output signal is proportional to the product of the amplitudes and the phase difference, it cannot be distinguished if they vary simultaneously; it requires an elaborate calibration process to measure the output quantitatively and the bandwidth of the signal measurements is limited by the characteristic frequency of the loop filter for the feedback control.

A TBI has two output ports. In general, for a Michelson interferometer (MI), since the output beam from one output port returns to the light source, the other open port is used as the detector port; while, for a Mach–Zehnder interferometer (MZI), both output ports are used as detection ports. Since the output signals are $180^{\circ }$ out of phase with respect to each other, the balanced detection scheme, in which the signals from the output ports are subtracted by using a high common-mode rejection differential amplifier, is used for doubling the signal strength and rejecting the intensity noise of the light source. Though it can provide very high sensitivity limited by photon noise—a fundamental quantum noise in the classical measurements—it not only requires a complicated feedback control loop but also has limited applications because, in some applications, phase and amplitude may vary simultaneously.

The homodyne in-phase and quadrature (I/Q) interferometer scheme has been developed to mitigate the drawbacks of a TBI [18,19,20,21,22]: it consists of two identical balanced homodyne interferometers (BHIs) with $90^{\circ }$ phase difference; thereby, the phase difference between two beams and the product of the corresponding amplitudes can be obtained independently and simultaneously by using the typical quadrature demodulation scheme. Any two-beam interferometer scheme, MI, MZI, or any variation of them, can be modified to have I and Q demodulation ports by manipulating the state of polarizations of the two beams and making use of polarization optics. Therefore, it can not only be used for conventional precision sensor applications but also has many new applications such as biosensors, bio-imaging, local complex reflectance analysis of an inhomogeneous surface, and so forth [20,21,22]. Another big advantage of employing an I/Q interferometer scheme is that it does not require any feedback control for locking the optical path length difference between two arms to keep it at a specific optimum condition.

Conventional homodyne I/Q interferometers, however, still have several practical drawbacks: (1) two balanced homodyne detectors require four photodiodes and careful optical alignment and balancing; (2) residual imbalance from non-ideal optical and electronic components can limit common-mode rejection; and (3) the overall configuration is difficult to integrate into a compact and robust system. Moreover, recent developments in precision interferometry have increasingly favored compact implementation, real-time digital signal processing, and reduced instrumental complexity [23,24,25,26], making such detection and alignment overhead more significant. Therefore, reducing the detection and implementation overhead while preserving the advantages of quadrature phase retrieval is desirable for modern interferometric sensing systems.

In this paper, we are proposing a new I/Q interferometer scheme, in which only two photodetectors, one in I-BHI and the other in Q-BHI, are used for measuring the phase difference and the product of amplitudes between the two beams of a TBI. It will be shown both theoretically and experimentally that, by taking the temporal difference between two consecutive output signals (hereafter referred to as the time differential signal (TDS)) from the corresponding photodiodes (PDs), DC bias intensity terms in the two output signals are removed and, hence, the resulting signals can be used as I and Q signals for I/Q demodulation. It will also be shown that the phase and amplitude change can be measured at the photon-noise limit, because any common mode noise including intensity noise from the imperfect light source is rejected in TDS. The reduced number of photodetectors simplifies the detection system by reducing the complexity associated with detector alignment and calibration. This advantage may be particularly significant for full-field imaging and multi-channel interferometric sensing, where a lower detector count and reduced readout complexity could facilitate more compact and practical implementations. The theory of TDS measurements and I/Q signal processing is presented in Section 2. The experimental setup used to validate the proposed concept is described in Section 3. Measurement results for both small and large amplitude displacements, along with a sensitivity analysis, are presented in Section 4. Finally, conclusions are drawn in Section 5.

## 2. Theory and Design Considerations

The output optical signal from one detection port of the I/Q interferometer—for example, the I-port—can be measured by using a photodiode. The number of output photons detected by the PD in a time interval Δt can be written as:

(1) $$\begin{array}{cc} N_{I}\left(t\right) & =\frac{\eta_{I}}{4}\left[N_{1}\left(t\right)+N_{2}\left(t\right)\right.\\ \; & \;\left.-2\sqrt{N_{1}\left(t\right)N_{2}\left(t\right)}\cos\phi (t)\right]+\sigma_{NI}\left(t\right), \end{array}$$

where ϕ(t) is the phase difference between the optical beams propagating along two different paths 1 and 2, $\eta_{I}$ is the quantum efficiency of the PD, $P_{1}=N_{1}\left(t\right)h\nu$ and $P_{2}=N_{2}\left(t\right)h\nu$ are optical power carried by beams 1 and 2, respectively, and hν is photon energy. A coherent light can be represented as a classical monochromatic electromagnetic field with a constant amplitude contaminated by photon noise. Therefore, for an ideal interferometer with a coherent input light, $P_{1}=P_{2}=P_{0}/2$, where $P_{0}$ is the optical power emitted by the coherent light source, which is constant over time. In this ideal case, the last term $\sigma_{NI}\left(t\right)\approx \sqrt{N_{I}/\Delta \nu }$, where Δν is the detection bandwidth, the photon noise, which is the fundamental quantum noise in optical detection. In practice, however, an imperfect light source may add power fluctuation noise mainly at low frequencies through the bias terms, the first two terms of the RHS of the Equation (1). As shown in the next section, it is possible to make another detection port—for example, Q-port—that is, in theory, identical to the I-port but has a $90^{\circ }$ phase difference. The output signal from PD in the Q-port is then given by

(2) $$\begin{array}{cc} N_{Q}\left(t\right) & =\frac{\eta_{Q}}{4}\left[N_{1}\left(t\right)+N_{2}\left(t\right)\right.\\ \; & \;\left.-2\sqrt{N_{1}\left(t\right)N_{2}\left(t\right)}\sin\phi (t)\right]+\sigma_{NQ}\left(t\right), \end{array}$$

where $\sigma_{NQ}\left(t\right)\approx \sqrt{N_{Q}/\Delta \nu }$ is the photon noise in the Q-port measurement. In the following calculations, we will assume that $\eta_{I}=\eta_{Q}\approx 1$, and the corresponding signal-to-noise ratio (S/N) for each measurement is sufficiently large that photon noise can be neglected. The interference caused by photon noise will be discussed quantitatively in conjunction with the actual measurement results.

Even though the third terms in Equations (1) and (2) have I and Q information, neither the phase difference nor the amplitudes of the interfering beams can be extracted directly without any calibrations because of the offset given by the first two bias terms in the RHS of Equations (1) and (2). In addition, the bias terms add low-frequency noises to the measurements that originate from power fluctuations of the light source. In a conventional 4-port I/Q demodulation scheme those bias terms are subtracted out by using the balanced detection scheme, for which the phase difference between two beams, ϕ(t), and the product of the amplitudes of the two beams, $\sqrt{N_{1}\left(t\right)N_{2}\left(t\right)}$, can be calculated simultaneously [27]. The 4-port scheme, however, adds complexities in both instrumentation and alignments of optical components. These complexities can be mitigated significantly if only 2-ports, one in I- and the other in Q-port, are used for measurements. The outputs from the corresponding ports can be represented by Equations (1) and (2). It will be shown that both the phase difference and the amplitude can be obtained by making use of the differential measurements between the temporally displaced signals in each port.

Signals $N_{I}\left(t\right)$ and $N_{Q}\left(t\right)$ are digitally sampled by using a high-speed analog-to-digital converter (ADC). It is a good approximation that a stabilized, single-frequency laser can be considered as a coherent light source and, thereby, the first two terms in Equations (1) and (2), which represent the total optical power of the light source, are approximately constant for at least many consecutive sampling periods. Therefore, if the phase difference between two consecutive signals at times *t* and $t-\tau_{d}$ is very small, i.e., $\Delta \phi \left(t\right)=\phi \left(t\right)-\phi \left(t-\tau_{d}\right)\ll 1$ then it can be shown that

(3) $$\begin{array}{cc} \Delta N_{I}\left(t\right) & =N_{I}\left(t\right)-N_{I}\left(t-\tau_{d}\right)\\ \; & \approx \frac{1}{2}\sqrt{N_{1}\left(t\right)N_{2}\left(t\right)}\Delta \phi \left(t\right)\sin\phi (t), \end{array}$$

where $\tau_{d}=\Delta t=n\tau_{s}$ is the time delay between two consecutive measurements, $\tau_{s}$ is the sampling time of the ADC, and *n* is the number of sampling intervals between them. Similarly, it can be shown that

(4) $$\begin{array}{cc} \Delta N_{Q}\left(t\right) & =N_{Q}\left(t\right)-N_{Q}\left(t-\tau_{d}\right)\\ \; & \approx -\frac{1}{2}\sqrt{N_{1}\left(t\right)N_{2}\left(t\right)}\Delta \phi \left(t\right)\cos\phi (t). \end{array}$$

Any correlated low-frequency intensity noise from the light source is canceled out in the differential signals given in Equations (3) and (4). Therefore, in a properly controlled environment with vibration isolation, acoustic shield, and so forth, the sensitivities of these measurements are limited by photon noises, which are increased by a factor of $\sqrt{2}$ relative to those of the corresponding single channels [11]. The phase difference between the two beams at t is given by

(5) $$\begin{array}{cc} \phi (t) & =-\;\arctan\left[\frac{\Delta N_{I}\left(t\right)}{\Delta N_{Q}\left(t\right)}\right]\\ \; & =-\;\arctan\left[\frac{N_{I}\left(t\right)-N_{I}\left(t-\tau_{d}\right)}{N_{Q}\left(t\right)-N_{Q}\left(t-\tau_{d}\right)}\right]. \end{array}$$

If $\phi (t-\tau_{d})$ is already measured in the previous measurement then the phase change can be calculated as

(6) $$\begin{array}{c} \Delta \phi \left(t\right)=\phi \left(t\right)-\phi \left(t-\tau_{d}\right) \end{array}$$

and, from Equation (3), it can be shown that

(7) $$\begin{array}{c} \sqrt{N_{1}N_{2}}=\left|\frac{N_{I}\left(t\right)-N_{I}\left(t-\tau_{d}\right)}{\Delta \phi (t)\sin\phi (t)}\right|. \end{array}$$

Therefore, we can measure the phase difference and the product of the amplitudes of the two beams of the interferometer.

It should be noted that as Δϕ(t)→0 during the measurements, photon noise may become significant in comparison to the signals. As an example, for the measurements of the mechanical motions of one of the mirrors in a TBI, Δϕ(t)=0 and, thereby, $\Delta N_{I}\left(t\right)=\Delta N_{Q}\left(t\right)=0$ at a turning point where the mirror is momentarily at rest. Therefore, there is no meaning in measuring ϕ(t) in the vicinity of a turning point. In this case, since Δϕ(t) may be too small to be measured in the classical limit, it is a good approximation to let $\phi \left(t\right)=\phi \left(t-\tau_{d}\right)$; one can set a threshold value for TDS measurements—for example, $\Delta N_{Th}$—and take this approximation when both $\left|\Delta N_{I}\left(t\right)\right|$ and $\left|\Delta N_{Q}\left(t\right)\right|$ become smaller than the threshold value. An adaptive delay scheme, for which the delay time $\tau_{d}$ is extended until the TDSs become larger than the threshold value, can also be adopted to skip the region where S/N<1. Alternatively, if a phase estimation is still required in the turning-point region, ϕ(t) can be calculated by using the following equation:

(8) $$\begin{array}{c} \phi \left(t\right)=\cos^{-1}\left[-\frac{\sqrt{2}\left(N_{I}\left(t\right)-N_{Q}\left(t\right)\right)}{\sqrt{N_{1}N_{2}}}\right]-\frac{\pi }{4} \end{array},$$

where $\sqrt{N_{1}N_{2}}$ can be calculated from Equation (7) by using the previous measurements before the turning-point region. In practice, this estimation may be obtained via a local average of the most recent valid measurement results.

## 3. Experimental Setup and Signal Processing

**Figure 1:**
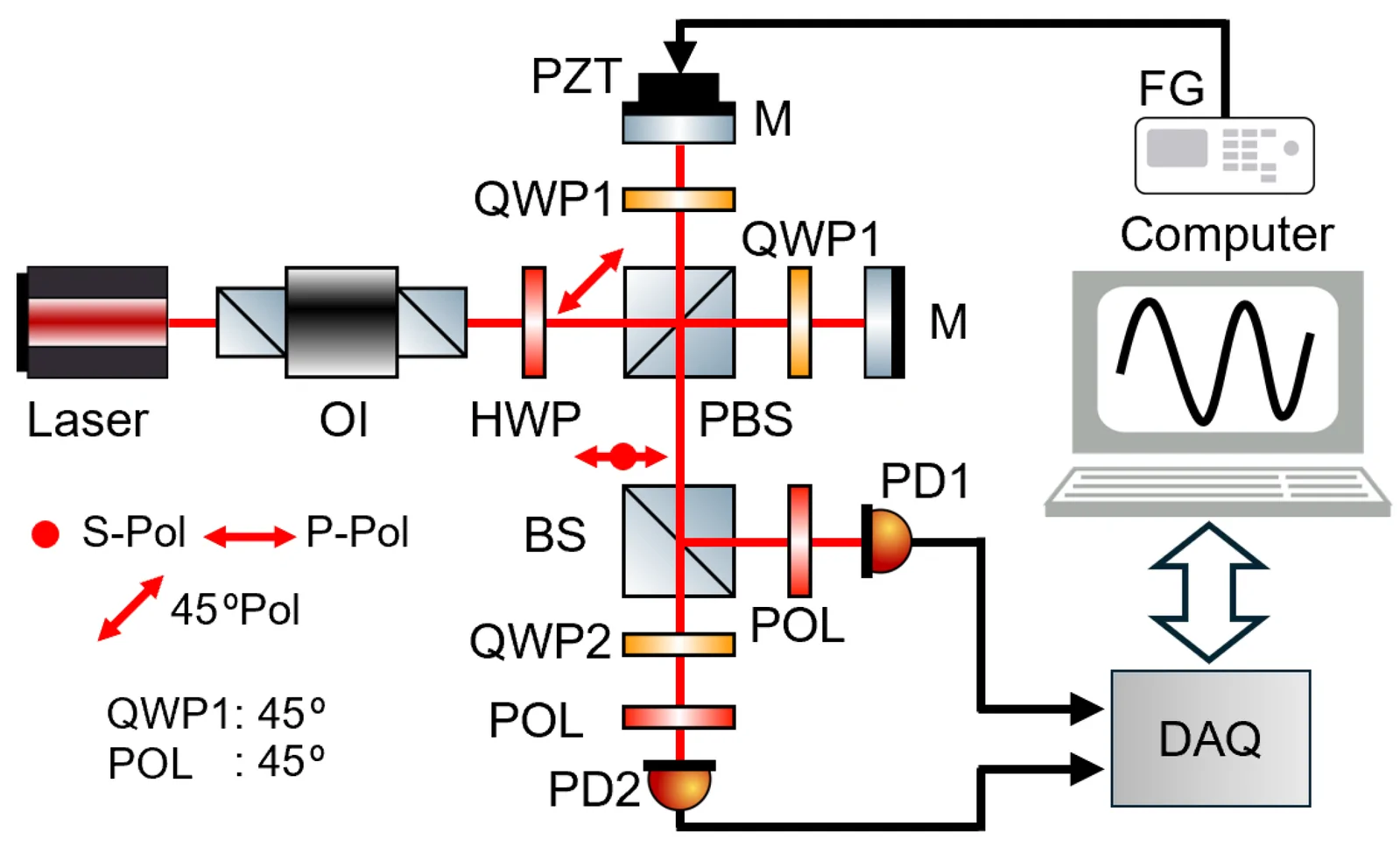
Schematic of experimental layout for the two-port phase-retrieval demonstration. In the figure, FG: function generator, POL: polarizer, OI: optical isolator, HWP: half-wave plate, QWP: quarter-wave plate, PBS: polarizing beam splitter, BS: beam splitter, PZT: piezo-electric transducer, PD: photo diode, DAQ: data acquisition, M: Mirror.

To prove the concept of the proposed two-port detection scheme, we used the polarization-based Michelson interferometer (PBMI), which used polarization optics to modify the beam paths as shown in Figure 1. A single frequency-stabilized He–Ne laser (HRS015, Thorlabs) was used for the light source. The plane of polarization (POP) of the laser beam was oriented at $45^{\circ }$ to the preferred axes of the polarizing beam splitter (PBS) by using a half-wave plate (HWP), so that the incident beam split into two different beams with equal intensities, which traveled along the corresponding arms of the PBMI and returned back to the PBS along the same path by using the mirror at the normal incidence angle. Each beam made a double pass through the quarter-wave plate (QWP1) placed in the beam path, in which the principal axes were oriented at $45^{\circ }$ to the POP of each propagating beam. The POPs of the returning beams were, thereby, rotated by $90^{\circ }$, and the combined beams exited to the other output port of the PBS. The co-propagating orthogonally polarized output beams were split into two readout ports by using a 50:50 beam splitter. A quarter-wave plate (QWP2), for which the principal axes were parallel to the POPs of the orthogonally polarized beams, was inserted in one path to meet the quadrature condition between the two readout ports. The two orthogonally polarized co-propagating beams were mixed by using the corresponding polarizer oriented at $45^{\circ }$ to the POPs. The interference signals of the readout ports were measured by using the photodiodes (SM05PDA2, Thorlabs). The resulting signals were then represented by Equations (1) and (2).

A mirror in one arm was mounted on a piezo-electric transducer (PZT) stage to apply a small, time-varying displacement to induce a controlled phase difference between the two arms of the interferometer. The output signals from PD1 and PD2 were acquired by using a PXI-4462 data acquisition module (National Instruments; 24-bit resolution, up to 204.8 kS/s per channel). Signal processing was performed offline for convenience, although real-time implementation is also feasible.

## 4. Results and Discussion

**Figure 2:**
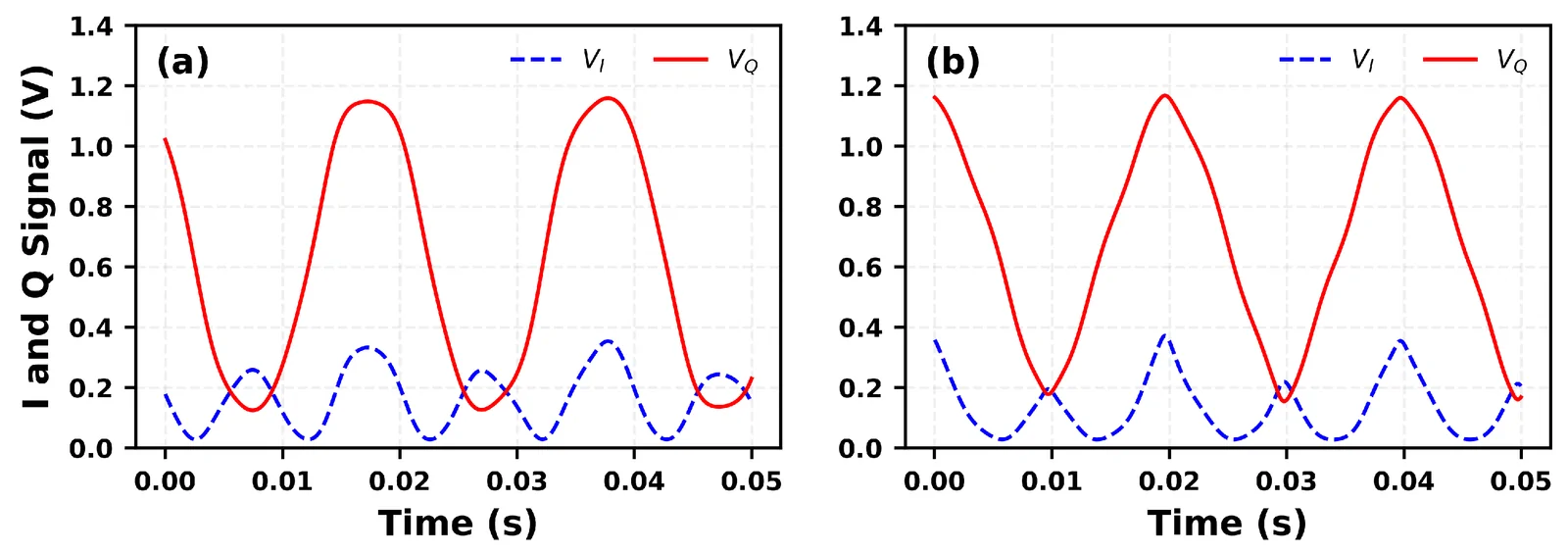
The output voltage signals $V_{I}$ from PD1 and $V_{Q}$ from PD2 for (**a**) sinusoidal and (**b**) triangular phase modulation. The relation between $V_{i}$ and $N_{i}$ is shown in Equation (9).

To show the feasibility of the proposed scheme, two different types of vibrations, one sinusoidal and the other a periodic triangular (PTA) vibration, with an equal peak-to-peak amplitude of ∼50nm and frequency of 50Hz were applied to the PZT mounted mirror. Sinusoidal and PTA modulations were selected because they provide distinctly different phase-change behaviors near the turning points. The sinusoidal waveform has a gradually vanishing slope, whereas the PTA waveform maintains an approximately constant slope except in the immediate vicinity of the turning points. This provided a direct comparison for the performance of the TDS phase retrieval under different local phase-change conditions. Archetypal output signals from PDs for sinusoidal and PTA modulations are shown in Figure 2a and Figure 2b, respectively. In both figures, the output voltage from PD1 and PD2 are shown as $V_{I}$ (blue trace) and $V_{Q}$ (red trace), which are proportional to $N_{I}\left(t\right)$ and $N_{Q}\left(t\right)$ through the following relationship:

(9) $$\begin{array}{c} V_{i}=\eta N_{i}eR_{L}=\eta \frac{P_{i}}{h\nu }eR_{L}=RP_{i}R_{L} \end{array},$$

where *i* represents *I* or *Q* port, $P_{i}$ is the optical power delivered to the PD in the *i* port, R is the responsivity of the PD, hν is photon energy, and $R_{L}$ is the load resistance of the transimpedance amplifier. In this experiment, R≈0.3A/W, $R_{L}=10\;k\Omega$, and the output power of the stabilized single-frequency He–Ne laser was $P_{0}\approx 1\;\mathrm{mW}$ or $N_{0}=N_{1}+N_{2}\approx 3.2\times 10^{15}/\sec$. Therefore, for a 1V signal the corresponding photon flux was $\approx 1.1\times 10^{15}/\sec$.

**Figure 3:**
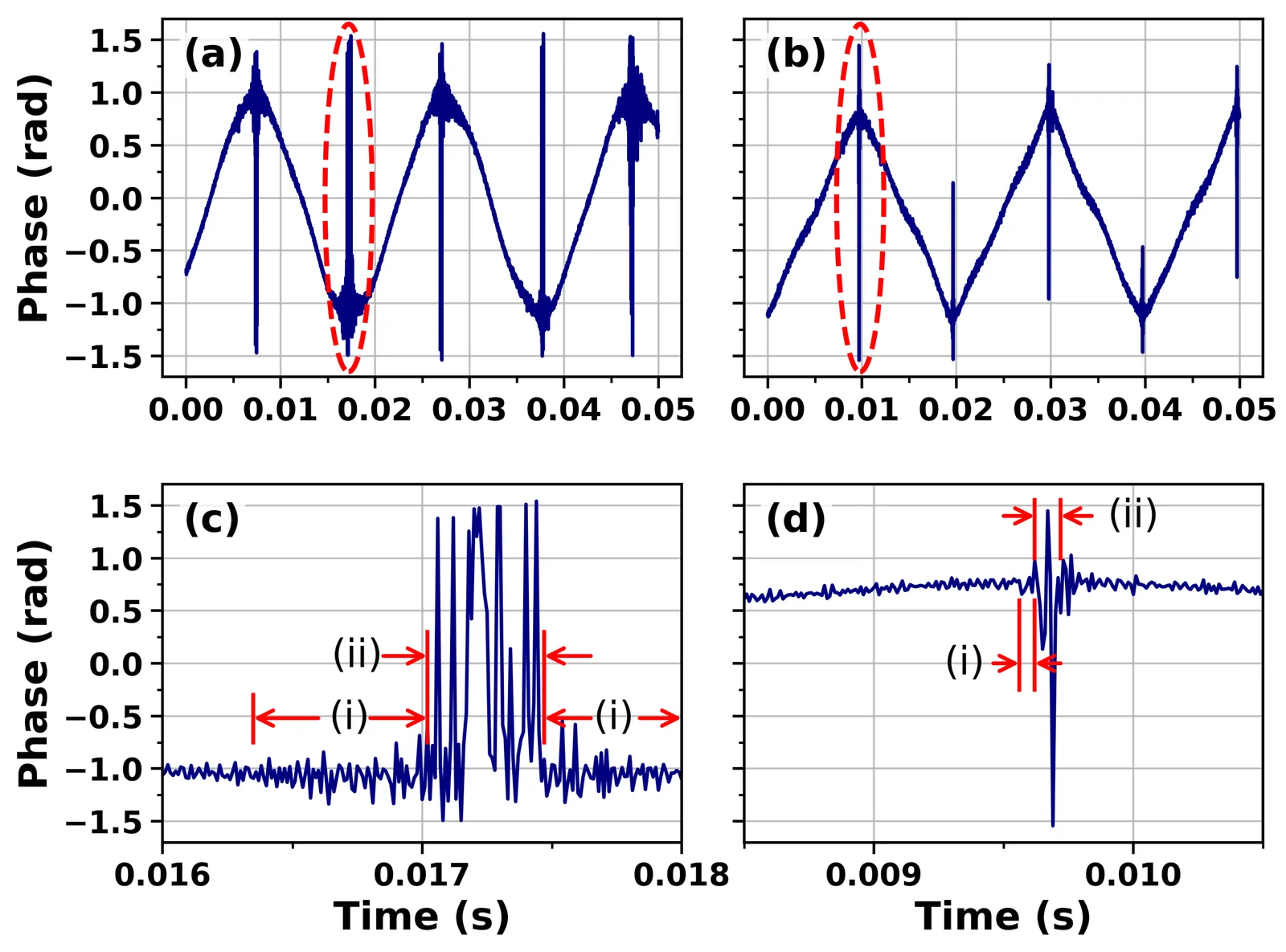
Measured phases for (**a**) sinusoidal and (**b**) PTA modulation. Enlarged views of the regions specified by dotted ellipses in (**a**) and (**b**) are shown in (**c**) and (**d**), respectively. It is evident from these figures that interference caused by photon noise is getting larger and the SNR becomes smaller in region (i). Noise becomes dominant over the signal and measured phase fluctuates over the full range of the arctangent, −π/2 to +π/2, in region (ii).

The I- and Q-port TDS signals, $\Delta N_{I}\left(t\right)$ and $\Delta N_{Q}\left(t\right)$, were obtained by using Equations (3) and (4) for the case of $\tau_{d}=\tau_{s}$, and the phases were calculated by using Equation (5). The results on the sinusoidal and PTA modulations are shown in Figure 3a and Figure 3b, respectively. It should be noted that the measured phase for the sinusoidal modulation had more noise in the vicinity of the turning points (TPs) than that for the PTA modulation, which is evident in the corresponding enlarged view of the signals, Figure 3c and Figure 3d, in the regions specified by a pair of arrows in Figure 3a and Figure 3b, respectively. In the following, we show that photon noise is responsible for these measurement characteristics in the vicinity of a turning point.

In the case of a sinusoidal modulation at frequency $\nu_{m}$, the phase difference between the two arms of the TBI can be written as

(10) $$\phi \left(t\right)=\phi_{m}\sin\left(2\pi \nu_{m}t\right)+\phi_{0},$$

where $\phi_{0}$ is the initial quasi-static phase difference between the two beams and $\phi_{m}$ is the amplitude of the phase modulation. If the measurement is performed at a moment near a TP, $t=t_{TP}-t_{\delta }$, where $t_{TP}$ is the time at the TP and $|t_{\delta }|\ll 1/4\nu_{m}$ is the time difference between the measurement and the TP, it can be shown that the phase change between two consecutive moments $t=t_{TP}-t_{\delta }$ and $t-\tau_{d}$ is, by using Equation (6), $\Delta \phi \left(t\right)\approx \phi_{m}{\left(2\pi \nu_{m}\right)}^{2}\left(2t_{\delta }+\tau_{d}\right)\tau_{d}/2$.

If the measurement is performed at the TP, i.e., if $t_{\delta }=0$ then the TDS signals, $\Delta N_{I}$ and $\Delta N_{Q}$, and thereby the corresponding signal-to-noise ratios (SNRs) are proportional to $\tau_{d}^{2}$. In our measurements, since $\nu_{m}=50$ Hz, $\tau_{d}=\tau_{s}=10$ µs, and $\phi_{m}\approx 1.0$, it can be shown that $\Delta \phi \left(t_{TP}\right)\approx 4.9\times 10^{-6}$. The actual experimental parameters that led to the values of the photon-noise-limited minimum detectable phase differences, ${\left(\Delta \phi \left(t_{TP}\right)\right)}_{\min}$, are given in Table 1, which shows that, in the vicinity of the turning points, the phase measurements were obscured by the corresponding photon noises.

If $t_{\delta }\neq 0$ but was close to the TP then Δϕ(t) was proportional to $\left(2t_{\delta }+\tau_{d}\right)\tau_{d}$. Since $t_{\delta }=l\tau_{s}$, where l=±1,±2,⋯, and $\tau_{d}=\tau_{s}$, SNR was proportional to $\left(2l+1\right)\tau_{s}^{2}$ and got larger linearly as *l* increased. Therefore, as $t_{\delta }$ got closer to a TP, the I- and Q-channel TDS signals became smaller and the corresponding photon noise became more significant, for which the SNRs were degraded, as can be seen in the two regions specified by (i) in Figure 3c. As $t_{\delta }$ got even more close to a TP, so that the noise became significantly larger than the signal, then the phase became unmeasurable and interference caused by photon noise increased rapidly to extremely large fluctuations ranging from −1.5rad. to 1.5rad. in the region specified by (ii) in Figure 3c. We believe that such large fluctuations were mainly due to dividing I-channel noise with Q-channel noise and taking the arctangent of it.

In the case of the PTA modulation, the magnitude of the corresponding phase difference between two consecutive moments *t* and $t-\tau_{d}$ was $\left|\Delta \phi \left(t\right)\right|=4\phi_{m}\nu_{m}\tau_{d}$, which is, for an ideal PTA, the same at any time except at TPs and is much larger than the photon noise. For an ideal PTA, Δϕ(t) must be discontinuous at a TP. However, in practice, the PZT actuator rounded off the abrupt change in slope because of the capacitance of the device, which acted as a high-pass filter. Therefore, regarding the nature of the PTA, in the range very close to a TP, it is a good approximation that $|\Delta \phi \left(t_{TP}\right)|\propto \phi_{m}\tau_{d}^{2}$. As in the case of sinusoidal modulation, photon noise became dominant in this region, which was much narrower than that of the sinusoidal modulation and in good agreement with the results shown in Figure 3b,d. These modulation-dependent noise characteristics in the vicinity of TPs become even clearer by comparing the widths and magnitudes of regions (i) and (ii) shown in Figure 3c,d.

**Figure 4:**
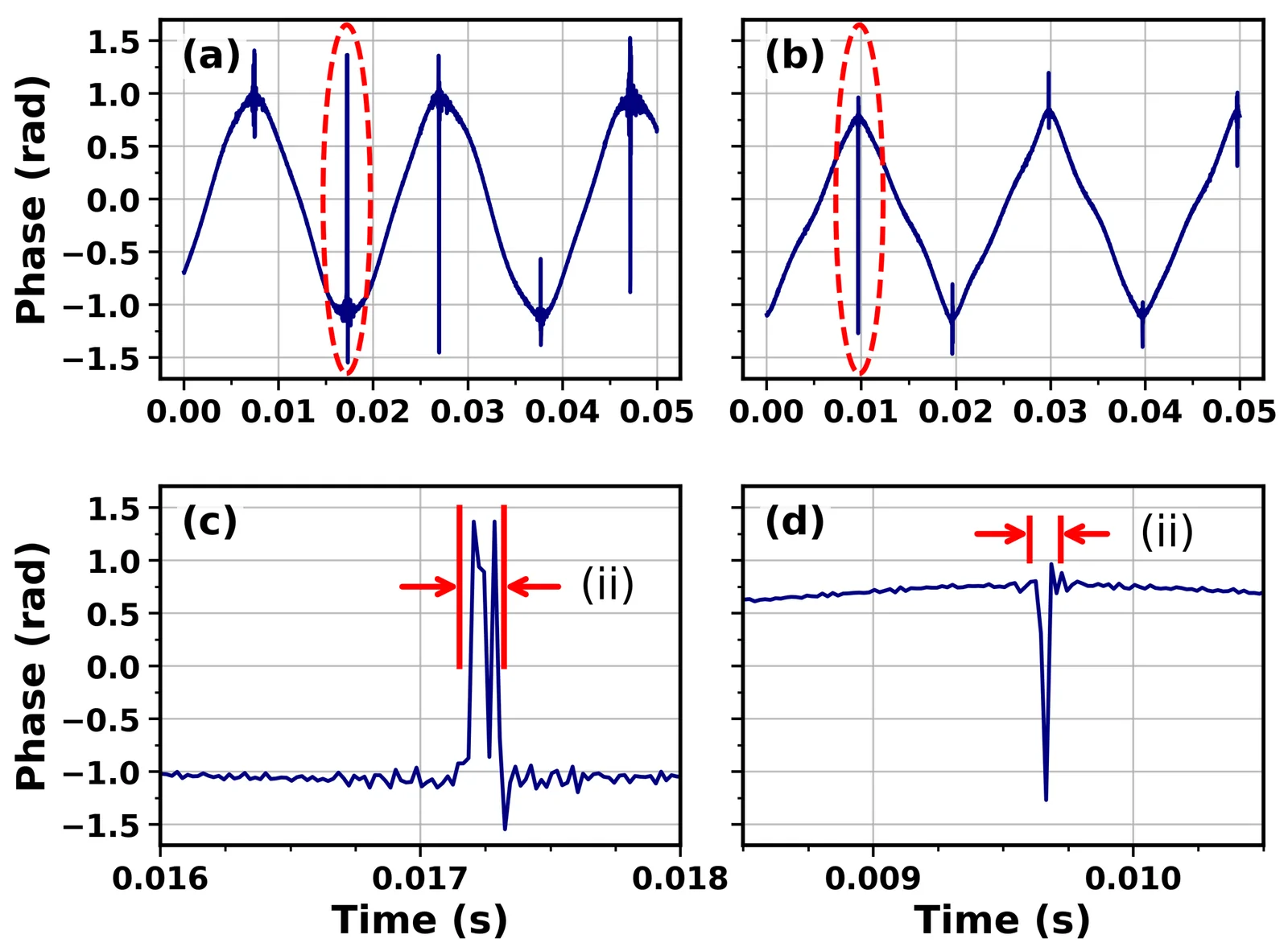
Phase measurements after applying a two-point tumbling average for (**a**) sinusoidal and (**b**) PTA modulations. Enlarged views of region (ii) near the turning points, indicated by the red dashed ellipses in (**a**) and (**b**), are shown in (**c**) and (**d**), respectively, where the phase fluctuations were reduced by the averaging.

The best way of improving SNR is increasing $\tau_{d}$, the temporal shift between two consecutive measurements, and the best way of increasing $\tau_{d}$ for a given sampling time $\tau_{s}$ may be tumbling average for output signals in the I- and Q-channel, in which $\tau_{d}=n\tau_{s}$, where *n* is the number of averages: this increases the signal by a factor of $n^{2}$ and removes noise with frequency higher than $1/\tau_{d}$. Since intensity noises in a stabilized single-frequency laser are originated from low-frequency environmental perturbations—such as a change in ambient temperature, low-frequency acoustic noise coupled into the laser resonator, and so forth—the limitation on $\tau_{d}$ is given by the coherence time of the light source, which was ∼1 ms for the laser used in this work. The phase measurements, after taking the tumbling average with n=2, for the cases of the sinusoidal and PTA modulations are shown in Figure 4a and Figure 4b, respectively, in which a significant improvement of the SNRs over the corresponding results in Figure 3a,b can be observed. The effectiveness of the tumbling average is even clearer to see in the extended view shown in Figure 4c,d, which show that, comparing them with the corresponding results in Figure 3c,d, not only the noise levels but also the widths of the large fluctuations were significantly reduced. As a result of these measurements and analysis, we found that the theoretical interpretation agrees well with the experimental results. Therefore, the TDS I/Q demodulation is a very useful phase demodulation scheme because it can not only measure the phase and the phase difference between two consecutive measurements with a simple optical arrangement but can also provide information on SNR and the moment of TP.

**Figure 5:**
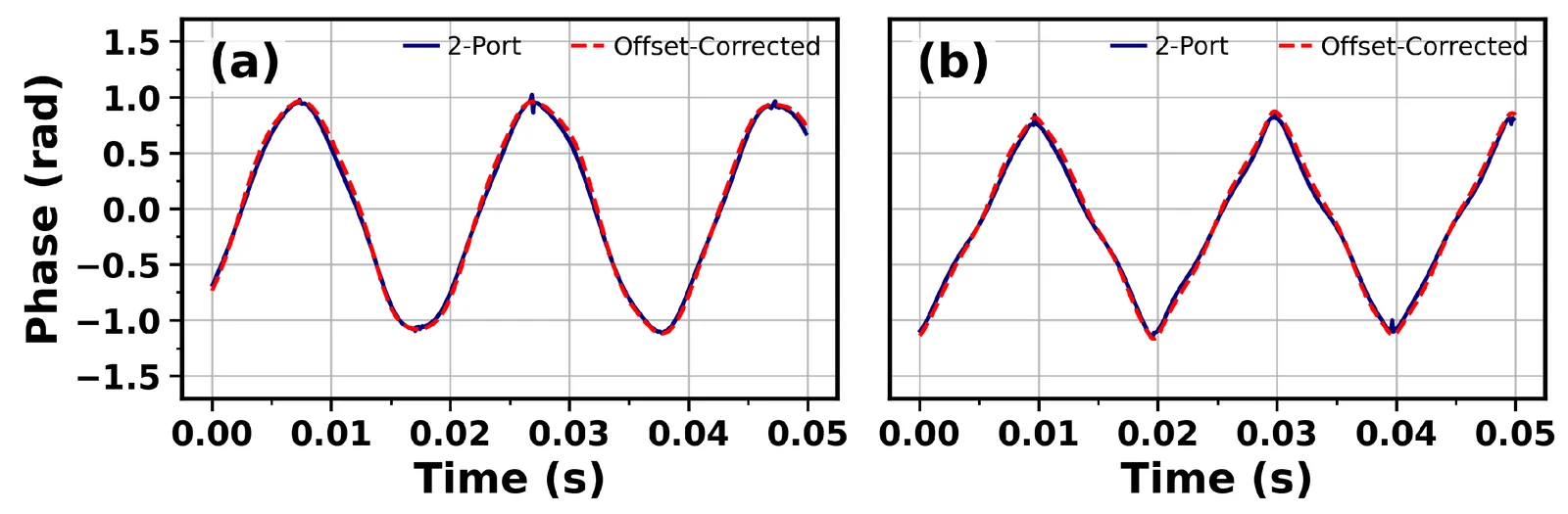
Comparison between the two-port phase reconstruction results after taking 10-point tumbling average (blue solid lines) and the conventional offset-calibrated (red dashed lines). The results for (**a**) sinusoidal and (**b**) triangular modulations are shown.

For the results shown in Figure 5, a 10-point tumbling average was applied. No significant fluctuations were observable for the sinusoidal and PTA modulations, shown as blue solid lines in Figure 5a and Figure 5b, respectively. In the results shown in Figure 3a,b, the measured signals were oversampled, so that the time difference between the two consecutive measurements, $\tau_{d}$, became very short in the vicinity of a TP. As a result, the TDS signals could become smaller than the photon noise, leading to large fluctuations in the retrieved phase. Increasing $\tau_{d}$ by the tumbling average suppressed these fluctuations significantly. As a reference, the results obtained using the conventional phase-retrieval method with offset calibration are shown as red dashed lines and are in good agreement with the proposed TDS I/Q demodulation results.

Minor mismatches can arise because the conventional reconstruction follows the Heydemann framework [28], but only the offset correction was applied in the present implementation. Quadrature-error correction was intentionally omitted because it would modify the reconstructed waveform and make it more difficult to verify the direct correspondence between the two results. Leaving the residual non-idealities uncorrected therefore allowed us to confirm that the proposed two-port reconstruction preserved the information contained in the raw readout signals.

**Figure 6:**
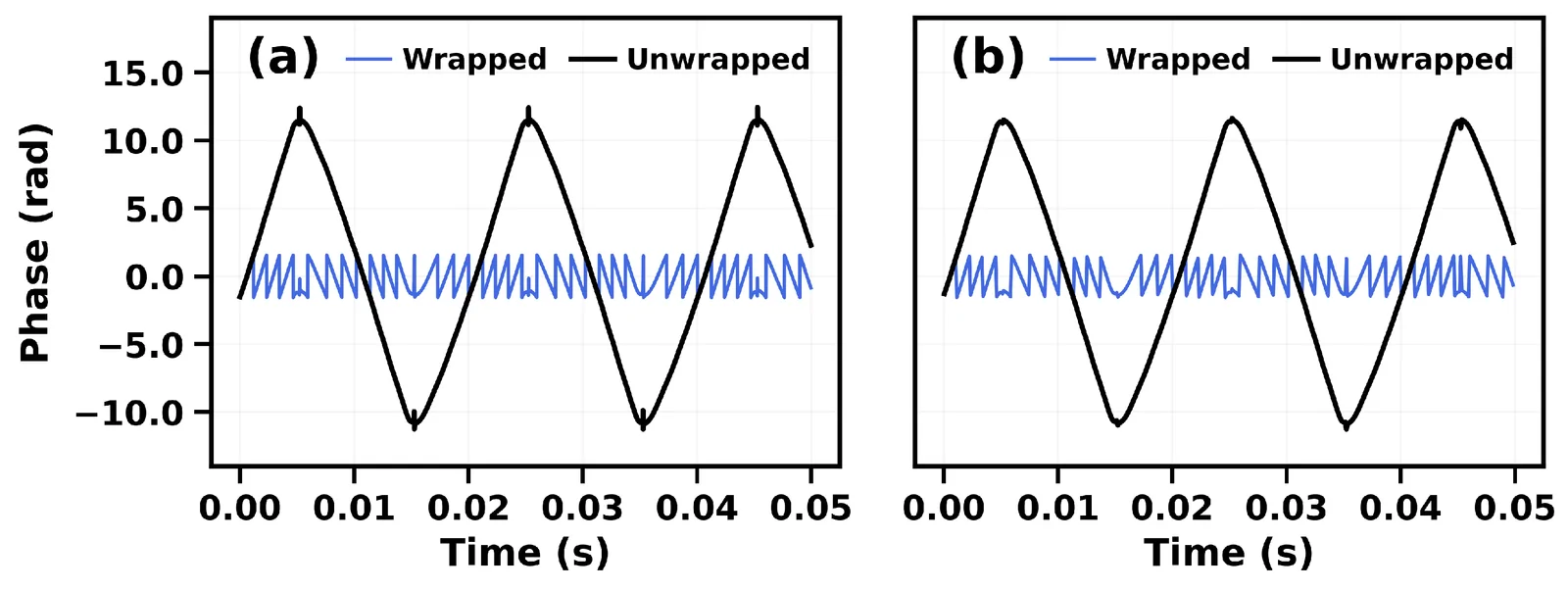
Measurement results for a ∼1.1 µm peak-to-peak PTA displacement. The phase difference was obtained by using TDS I/Q demodulation with (**a**) no average and (**b**) a 10-point tumbling average applied to $N_{I}$ and $N_{Q}$. In the figures, wrapped and unwrapped phases are shown as blue and black lines, respectively.

As another example, the TDS I/Q demodulation scheme was applied to a wide dynamic range measurement. The PZT mounted mirror was driven by a PTA signal to provide ∼1.1 µm (∼22 rad) peak-to-peak displacement at a 50Hz repetition rate, which provided TDS signals larger than photon noise except for the immediate vicinity of TPs. Figure 6 shows the directly demodulated phase (blue) and the corresponding unwrapped phase (black) for (a) no averaging and (b) a 10-point tumbling average. In contrast to the small-amplitude measurements discussed above, the larger phase excursion provided sufficiently large TDS signals over most of the waveform, including the vicinity of the TPs. Consequently, the phase could be continuously retrieved over multiple 2π cycles without loss of the induced phase information. The agreement between the unwrapped results obtained with and without averaging also indicates that the tumbling average suppressed local fluctuations without compromising the wide-range phase reconstruction.

## 5. Conclusions

We have shown both theoretically and experimentally that the phase at one moment, ϕ(t), can be measured by processing the differences between two consecutive signals at *t* and $t-\tau_{d}$ from one PD in I-port and the other PD in Q-port. To show the potential of the proposed scheme, the TDS I/Q phase demodulation scheme was applied for measuring three different types of phase modulations: A sinusoidal and PTA phase modulation with the same modulation amplitude, ∼0.1rad, and the PTA with a much larger modulation amplitude, ∼22rad, for which the modulation frequencies of these three types were the same at ∼50Hz. In the case of the small amplitude modulations, the corresponding SNRs grew smaller as the measurement time approached a TP. It has been shown that if the signals were oversampled then the time difference between consecutive measurements, $\tau_{d}$, was so small that in both cases of sinusoidal and PTA modulation the photon noises dominate the TDS signals and phases could not be determined in the vicinity of the TPs. As a simple solution, in the vicinity of turning points or in the region where I- and Q-channel signal changes become smaller than the prescribed level, unreliable phase estimates could be excluded. As another way, we showed theoretically that ϕ(t) can be calculated directly from Equation (8) by using the parameters obtained from the measurements in the region above the prescribed threshold. As we showed both theoretically and experimentally, the best way of solving this problem related to SNR is by adjusting the time delay between the consecutive measurements $\tau_{d}$. For a given sampling time $\tau_{s}$, we showed that taking the tumbling average not only increases the time delay by $\tau_{d}=n\tau_{s}$, where *n* is the number of data points on average, but also filters out the noise at frequencies higher than $1/\tau_{d}$. The results demonstrate that these approaches can suppress large phase fluctuations near turning points while preserving phase information. Since the bias terms are removed from the corresponding interference signals measured in each PD for obtaining TDS signals, the proposed scheme can provide an excellent common-mode rejection that can effectively remove the intensity noise of the light source. It should be noted that only two PDs are used for the I/Q demodulation of the phase and amplitude in the interferometric measurements by replacing conventional spatial balanced detection with temporal differential detection: reducing the number of PDs offers advantages in terms of compactness and implementation simplicity, which are crucial in actual sensor applications. In summary, we have shown that all the drawbacks of a conventional homodyne I/Q-interferometer scheme that were mentioned in the introduction have been greatly mitigated in the proposed scheme. In this context, the proposed two-detection-port scheme with a TDS processing algorithm can be implemented in many applications. It may be particularly attractive for emerging quantum sensing architectures, where experimental overhead often increases rapidly with detector count.

## Figures and Tables

**Table 1:** Photon number at photodiode.

	Turning Points (rad.)	Photon Number at the PD *	Photon Number in TDS *	Photon Noise	$\Delta \phi_{\min}$ (rad.) **
I-Channel	0.94	$3.3\times 10^{9}$	$6.5\times 10^{9}\Delta \phi$	$8.1\times 10^{4}$	$5.8\times 10^{-5}$
	−1.08	$4.2\times 10^{9}$	$7.1\times 10^{9}\Delta \phi$	$9.2\times 10^{4}$	$1.3\times 10^{-5}$
Q-Channel	0.94	$1.5\times 10^{9}$	$4.7\times 10^{10}\Delta \phi$	$5.5\times 10^{4}$	$1.2\times 10^{-5}$
	−1.08	$1.5\times 10^{10}$	$1.7\times 10^{11}\Delta \phi$	$1.7\times 10^{5}$	$1.0\times 10^{-6}$

* Photon numbers were calculated at the corresponding TPs during the measurement time interval $\tau_{s}=10^{-5}\;s$. ** Estimated for the detection bandwidth $\Delta \nu =10^{5}\;\mathrm{Hz}$.

## Data Availability

The datasets are available from the authors upon reasonable request.
